# Oral Cholera Vaccine Coverage, Barriers to Vaccination, and Adverse Events following Vaccination, Haiti, 2013^1^

**DOI:** 10.3201/eid2106.141797

**Published:** 2015-06

**Authors:** Rania A. Tohme, Jeannot François, Kathleen Wannemuehler, Preetha Iyengar, Amber Dismer, Paul Adrien, Terri B. Hyde, Barbara J. Marston, Kashmira Date, Eric Mintz, Mark A. Katz

**Affiliations:** Centers for Disease Control and Prevention, Atlanta, Georgia, USA (R.A. Tohme, K. Wannemuehler, P. Iyengar, A. Dismer, T.B. Hyde, B.J. Marston, K. Date, E. Mintz);; Ministry of Public Health and Population, Port-au-Prince, Haiti (J. Francois, P. Adrien);; Centers for Disease Control and Prevention, Port-au-Prince (M.A. Katz)

**Keywords:** oral cholera vaccine, coverage survey, Haiti, vaccination campaigns, cholera, bacteria

## Abstract

In 2013, the first government-led oral cholera vaccination (OCV) campaign in Haiti was implemented in Petite Anse and Cerca Carvajal. To evaluate vaccination coverage, barriers to vaccination, and adverse events following vaccination, we conducted a cluster survey. We enrolled 1,121 persons from Petite Anse and 809 persons from Cerca Carvajal, categorized by 3 age groups (1–4, 5–14, >15 years). Two-dose OCV coverage was 62.5% in Petite Anse and 76.8% in Cerca Carvajal. Two-dose coverage was lowest among persons >15 years of age. In Cerca Carvajal, coverage was significantly lower for male than female respondents (69% vs. 85%; p<0.001). No major adverse events were reported. The main reason for nonvaccination was absence during the campaign. Vaccination coverage after this campaign was acceptable and comparable to that resulting from campaigns implemented by nongovernmental organizations. Future campaigns should be tailored to reach adults who are not available during daytime hours.

## Abstract 2

Coverage was comparable to or higher than that in other countries.

Since October 2010, Haiti has endured one of the largest cholera epidemics ever recorded in a single country, accounting for 54% of all cholera cases and 41% of all cholera deaths reported to the World Health Organization (WHO) during 2010–2013 (*1*–*4*). Contributing to this sustained, ongoing epidemic were inadequate drinking water and sanitation infrastructure, worsened by the 2010 earthquake, and an immunologically naive population. In February 2013, the Haiti Ministry of Health and Population launched the 2013–2022 national plan of action for elimination of cholera (*5*). The plan outlined long-term interventions such as improving water quality, sanitation, and waste management. However, because these interventions will require years to implement, the Haitian government proposed vaccinating 600,000 persons during 2013–2015 as a short-term approach to help control the cholera epidemic (*6*). This decision was consistent with World Health Assembly Resolution 64.15, which calls for implementation of an integrated and comprehensive approach to cholera control that includes the use of oral cholera vaccine (OCV) (*7*).

OCVs are increasingly being used as part of preemptive and reactive vaccination strategies (*8*–*18*). Before 2011, Dukoral vaccine (Crucell, Stockholm, Sweden), licensed for use in persons >2 years of age (2 doses given 7 days to 6 weeks apart), was the only available WHO-prequalified vaccine approved for purchase by United Nations agencies on the basis of safety and efficacy. However, its use in vaccination campaigns was limited by the need to mix the vaccine in a buffer solution diluted in clean water and by its relatively high cost (US$3–6/dose). In September 2011, Shanchol vaccine (Shantha Biotechnics, Hyderabad, India) was prequalified by WHO (2 doses given 14 days apart). Shanchol offered several advantages over Dukoral, including approval for use in persons >1 year of age, administration without buffer or water, and lower price (US$1.85/dose). Recent data from Kolkata, India, indicated that the 5-year protective efficacy of 2 doses of Shanchol was 65% (95% CI 52%–74%) (*19*), and effectiveness 6 months after a vaccination campaign for outbreak control in Guinea was 86% (95% CI 56.7%–95.8%) (*20*). These findings further support the use of OCV in response to epidemic and endemic cholera.

In 2012, the first pilot OCV campaign was conducted in Haiti by 2 Haitian nongovernmental organizations (NGOs) in a rural area in Artibonite Department (target population for vaccination 50,000) and in an urban area in Port-au-Prince (target population 69,185) (*12*,*13*). In 2013, the Haiti Ministry of Health and Population conducted the first government-run OCV campaign as part of the national plan for the elimination of cholera. Shanchol was used, and the target population included (per manufacturer recommendations) persons >1 year of age, with the exception of pregnant women. Because only 200,000 doses of the vaccine were available, the Ministry of Health and Population chose to target Petite Anse, an urban area in the commune of Cap Haitian in the North Department (estimated target population 86,989), and Cerca Carvajal, a rural area in the Centre Department (estimated target population 20,917). These areas were chosen because they had the required target population for the available OCV doses, poor water and sanitation infrastructure, difficult access to health care services, and historically high cholera attack rates (10.1%–37%) (*21*) (Figure). The first vaccination round was conducted August 5–9, 2013. The second round was conducted August 26–30 in Cerca Carvajal and was split between August 26–28 and September 9−10 in Petite Anse because of depleted vaccine supplies and the time needed to receive additional doses. The campaign was conducted at fixed and mobile sites and through house-to-house visits. Vaccination cards specific for the campaign were used to document vaccination. Printed pamphlets including information about water, sanitation, and hygiene (WASH) and the need to receive 2 doses of the vaccine were distributed during the campaign. However, messages delivered orally varied between areas and included no details about the vaccine. Administrative coverage (coverage reported by the country) with 2 OCV doses was 92% in Petite Anse and 104% in Cerca Carvajal. Previously, administrative vaccination coverage estimates have been shown to be unreliable in Haiti because the number of persons in the target populations was not always known (*22*,*23*).

**Figure:**
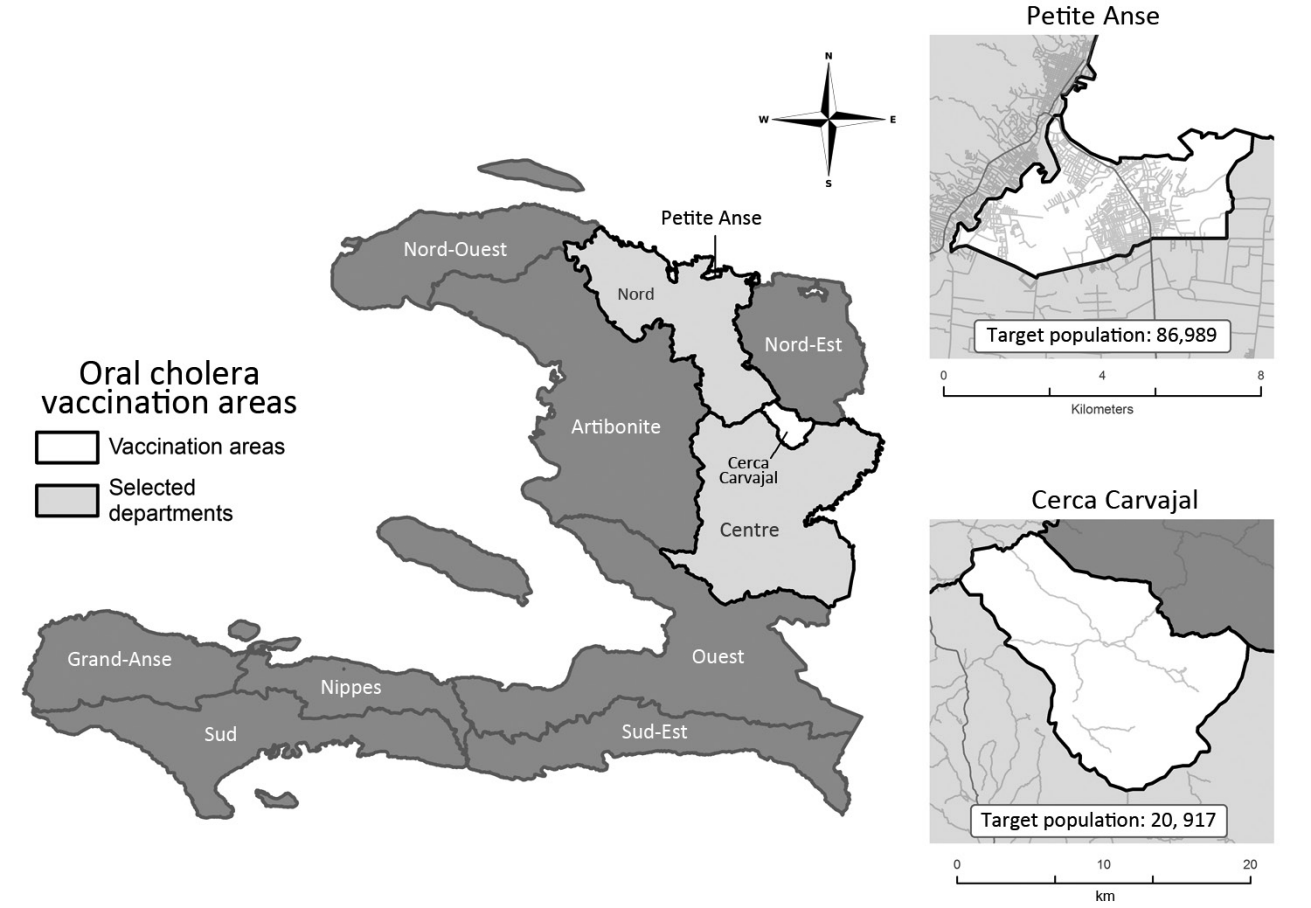
Areas selected for the first government-implemented oral cholera vaccination campaign in Haiti, 2013. Data source: Haiti Ministry of Health and Population, Centre National de l’Information Géo-Spatiale, and Institut Haïtien de Statistique et d’Informatique, OpenStreetMap.

To inform planning for future OCV campaigns in Haiti and other countries, we conducted a vaccination coverage survey. Compared with use of administrative coverage results, this method enables better assessment of the success of vaccination campaigns (evaluation of vaccine coverage, barriers to vaccination, and adverse events reported following vaccination).

## Methods

### Sampling and Study Population

We conducted a multistage cluster survey by using the 2011 household and population estimates provided by the Haitian Institute of Statistics and Information. The sampling frame consisted of 116 enumeration areas in Petite Anse and 25 in Cerca Carvajal. Enumeration areas are the primary sampling units, clearly delineated and mapped by the census bureau in Haiti, and are used as a sampling frame for major surveys in Haiti, including the Demographic and Health Survey. Sample size was calculated to estimate coverage by age group (1–4, 5–14 and >15 years) by using the following assumptions: 1) a desired precision of ±0.05, 2) an expected 2-dose OCV coverage of 85%, 3) a child 1–4 years of age in 65% of households, 4) a nonparticipation rate of 5%, and 5) a design effect of 1.7 in Petite Anse and 1.5 in Cerca Carvajal. These age groups were chosen for the purpose of comparison with OCV surveys conducted in other countries (*8*,*9*,*18*,*24*). A total of 564 households were needed in Petite Anse, and 353 households were needed in Cerca Carvajal.

We selected 30 (26%) enumeration areas from Petite Anse and 18 (72%) from Cerca Carvajal by systematic random sampling without replacement. In each enumeration area, 20 households were selected by systematic sampling. Finally, in each selected household, 1 person in each age group, if available, was randomly selected (by use of a random number table) for interview.

A household was defined as a group of persons who ate together and lived under the same roof. Persons in households were eligible to participate if they 1) were >1 year of age and not pregnant during the OCV campaign, 2) resided in a selected household during the OCV campaign, and 3) gave oral consent (for participants <18 years of age, consent was provided by a responsible adult member of the household). Responses for children were provided by the mother and the child if child was >5 years of age.

The protocol was approved by the national ethics committee in Haiti. It was classified as a program evaluation activity by the Centers for Disease Control and Prevention.

### Data Collection

Team members (2 interviewers and 1 supervisor) who had experience with Demographic and Health Surveys were trained on survey and household selection methods, interviewing, use of smartphones for data collection, and use of global positioning system units. The survey was conducted during September 13–27, 2013, <1 month after campaign completion.

Interviews were initiated with the first household in 1 of the corners of the enumeration area noted on the global positioning system device. Subsequent households were selected by using the systematic method of traversing the enumeration area by moving in a clockwise manner and skipping households according to a precalculated sampling interval (estimated total number of households in cluster divided by 20). Contact was initiated with an adult, usually the female head of household, who was interviewed by use of a standardized questionnaire. Information collected included general household information: access to treated water and health care facilities, previous history of cholera infection in the household, awareness of the OCV campaign, and the number of eligible persons in the household who were vaccinated with OCV during the campaign and the number of doses received. Next, for each household, 1 person was randomly chosen from each of the 3 age groups for an in-depth interview (for younger children, the mother provided the information). Each interview collected information about the interviewee’s age, sex, previous history of cholera, number of OCV doses received during the campaign (documented by card or by recall if the card was not available), vaccine administration (route, location, and whether person spat out the vaccine), adverse events within 14 days of receipt of the first and second OCV dose, and general knowledge about OCV (duration of protection, need for other measures for protection against cholera). Those who had not received the first or second dose were asked their reasons for not being vaccinated. Interviewers asked all questions without prompting for answers. Interviews were conducted in Haitian Creole, and answers were recorded on smartphones.

Households were visited at least 2 times if no one was at home or if a randomly selected person was unavailable during the first visit. Selected households were not replaced if they were not eligible or if no one was at home (no response) after at least 2 attempted visits.

### Statistical Analyses

Estimated percentages and 95% CIs were calculated by using SAS-callable SUDAAN version 10.01 (RTI International, Research Triangle Park, NC, USA) to account for the finite population at the first stage cluster sampling. Statistical weights for each household were based on the sampling probabilities of the first 2 stages, and statistical weights for each person were based on the sampling probabilities of all 3 stages. For each area, we estimated 1- and 2-dose OCV campaign coverage (including 95% CI) by age group and sex. Satterthwaite-adjusted χ^2^ tests were used to compare coverage between subpopulations. For each area we also calculated rates of dropout between receipt of first and second vaccine doses, reports of any adverse events, and reasons for not receiving vaccine.

## Results

### Household Characteristics

Of 960 visited households, 925 (96%) consented to participate (568 in Petite Anse, 357 in Cerca Carvajal). Of the participating households, 79% in Petite Anse and 46% in Cerca Carvajal were within a 15-minute walking distance of a drinking water source. In Petite Anse, the most common source of drinking water was bottled water or water purchased from a company (84%); in Cerca Carvajal, it was unprotected spring water (42%) and public piped water (34%).

For 56% and 21% of households in Petite Anse and Cerca Carvajal, respectively, the closest heath facility was located within 30 minutes of travel by the mode of transportation available in the household (walking, driving, motorcycle, or other). Overall, for 11% and 59% of the households in Petite Anse and Cerca Carvajal, respectively, the nearest health facility was >1 hour away. For ≈16% and 27% of households in Petite Anse and Cerca Carvajal, respectively, at least 1 household member had been infected with cholera during the past 2 years. At least 1 person had died of cholera in ≈3% of households (3.4% in Petite Anse and 3.2% in Cerca Carvajal).

### Campaign Awareness and Vaccination of Household Members

Of the 568 households in Petite Anse and 357 in Cerca Carvajal, 511 (91%, 95% CI 87%–93%) and 335 (93%, 95% CI 89%–96%), respectively, were aware of the 2013 OCV campaign. Of those who were aware, the principal sources of information were social mobilizers who used megaphones, followed by health care workers and friends/family. In Petite Anse, 79.9% (95% CI 75.5%–83.7%) of households had at least 1 eligible person who had received 2 OCV doses; in Cerca Carvajal, 89% (95% CI 83%–93%) of households had at least 1 person who had received 2 doses. All eligible household members had received 2 OCV doses in 23% (95% CI 17.4%–28.7%) of households in Petite Anse and 37% (95% CI 31%–44%) of households in Cerca Carvajal. 

### Vaccination among Enrolled Household Members

A total of 1,121 and 809 persons in Petite Anse and Cerca Carvajal, respectively, who were eligible for vaccination were enrolled and categorized into 1 of the 3 age groups (Table 1). Overall, 62.5% (95% CI 57.9%–66.9%) of eligible persons from selected households in Petite Anse and 76.8% (95% CI 71.1%–81.8%) from Cerca Carvajal received both doses of OCV (Table 2). Of those who received 2 doses, 51% from Petite Anse and 70% from Cerca Carvajal had card documentation of both doses. In Petite Anse, the dropout rate between the first and second OCV dose was 9.6% (95% CI 7.1%–12.9%) and was significantly higher among persons >15 years of age (12.0%) than among children 1–4 years of age (3.4%; p = 0.008). In Cerca Carvajal, the dropout rate between the first and second OCV dose was 8.4% (95% CI 5.5%–12.6%) and was significantly higher among male than female respondents (12.6% vs. 4.5%; p = 0.002). For both regions, 2-dose coverage was significantly lower among persons >15 years of age than among younger persons (p<0.01). In Cerca Carvajal, coverage was significantly lower among male than among female respondents overall (69.0% vs. 84.9%; p<0.001), among those 5–14 years of age compared with those in other age groups (76.5% vs. 92.9%; p = 0.005), and among those >15 years of age compared with those in other age groups (57.9% vs. 82.7%; p<0.001).

**Table 1:** General characteristics of participants in oral cholera vaccine coverage survey, Haiti, 2013

Characteristic	Area	
	Petite Anse, n = 1,121	Cerca Carvajal, n = 809
Sex, no. (%)		
M	499 (43.1)	407 (50.6)
F	622 (56.9)	402 (49.4)
Age, y, no. (%)		
1–4	206 (10.3)	192 (13.8)
5–14	353 (24.8)	263 (35.0)
>15	562 (64.9)	354 (51.1)
History of cholera, % (95% CI)	38 3.5 (2.4–5.2)	48 6.6 (4.5–9.6)

**Table 2:** Estimated oral cholera vaccination coverage, Haiti, 2013*

No. doses Received	Area, % (95% CI)						
	Petite Anse				Cerca Carvajal		
	Total, n = 1,118	Male, n = 497	Female, n = 621		Total, n = 808	Male, n = 407	Female, n = 401
Total							
2	62.5 (57.9–66.9)	59.8 (53.9–65.5)	64.5 (58.8–69.7)		76.8 (71.1–81.8)	69.0 (60.4–76.4)	84.9 (80.0–88.8)
1	6.6 (4.9–8.9)	7.1 (4.7–10.4)	6.3 (3.8–10.2)		7.0 (4.7–10.5)	10.0 (6.3–15.4)	4.0 (2.4–6.8)
0	30.9 (26.8–35.3)	33.1 (27.6–39.1)	29.2 (24.8–34.1)		16.1 (12.4–20.6)	21.1 (15.3–28.3)	11.0 (7.9–15.2)
Age group, y							
1–4	n = 206	n = 102	n = 104		n = 191	n = 91	n = 100
2	67.9 (60.2–74.8)†	63.9 (52.8–73.6)	71.8 (62.5–79.5)		81.6 (72.9–88.0)†	87.5 (75.5–94.1)	76.1 (63.1–85.6)
1	2.4 (0.8–6.9)	4.5 (1.4–13.4)	0.3 (0.0–2.4)		5.8 (2.7–12.0)	3.5 (0.8–13.9)	7.9 (3.2–18.5)
0	29.7 (23.3–37.0)	31.6 (22.3–42.5)	27.9 (20.3–37.1)		12.6 (7.9–19.6)	9.1 (4.1–18.8)	16.0 (9.1–26.5)
5–14	n = 351	n = 163	n = 188		n = 263	n = 148	n = 115
2	77.9 (71.7–83.0)†	75.5 (65.1–83.6)	79.8 (72.4–85.6)		83.8 (75.0–89.9)†	76.5 (63.6–85.9)	92.9 (85.1–96.7)
1	5.8 (3.7–9.0)	6.1 (3.1–11.3)	5.6 (3.0–10.3)		5.5 (2.8–10.8)	8.5 (4.0–17.2)	1.8 (0.6–5.5)
0	16.3 (12.1–21.7)	18.4 (11.5–28.2)	14.6 (9.8–21.2)		10.7 (6.2–17.7)	15.0 (8.3–25.5)	5.4 (2.0–13.7)
>15 y	n = 561	n = 232	n = 329		n = 354	n = 168	n = 186
2	55.7 (50.0–61.3)†	52.5 (44.8–60.0)	58.0 (50.7–65.0)		70.8 (63.9–76.9)†	57.9 (47.4–67.8)	82.7 (75.7–88.0)
1	7.6 (5.3–10.9)	7.9 (4.5–13.6)	7.4 (4.0–13.2)		8.4 (5.3–13.1)	12.9 (7.6–21.1)	4.3 (2.1–8.6)
0	36.7 (31.5–42.1)	39.6 (32.4–47.2)	34.6 (28.7–41.0)		20.8 (15.7–27.0)	29.2 (20.5–39.7)	13.0 (8.6–19.2)

*Vaccination status was assessed from special cards distributed to document doses administered during the campaign, if available, or by recall; in every selected household, 1 person was randomly selected from each age group. Statistical analyses accounted for the weights and the study design.  †Design effect (DE) and estimated intraclass correlations (ICC) = (DE−1)/(b−1), where b is the average number of responses per cluster. DE is based on accounting for clustering only (The finite population correction and weighting are ignored). Petite Anse: age group: 1–4 y, DE = 1.3 and ICC = 0.05; 5–14 y, DE = 1.4 and ICC = 0.04; >15 y, DE = 2.1 and ICC = 0.06. Cerca Carvajal: age group 1–4 y, DE = 2.4 and ICC = 0.15; 5–14 y, DE = 3.9 and ICC = 0.22; >15 y; DE = 2.3 and ICC = 0.07.

In Petite Anse, two thirds of respondents reported having received OCV at home (66.4%, 95% CI 58.5%–73.5%) and nearly a quarter at mobile posts (23.9%, 95% CI 18.1%–30.8%). In Cerca Carvajal, almost half of respondents reported having received OCV at mobile posts (49.6%, 95% CI 41.5%–57.8%) and 19.4% (95% CI 15.3%–24.3%) at health centers. About 7% and 5% of respondents in Petite Anse and Cerca Carvajal, respectively, reported spitting out part of the first dose because of its bad taste; <5% in both areas reported spitting out part of the second OCV dose.

### Knowledge about OCV

Of 1,459 respondents who had received at least 1 OCV dose, almost one third (34% in Petite Anse, 33% in Cerca Carvajal) reported that they thought OCV alone was enough to protect them from cholera. Most (73%) respondents did not know the duration of protection provided by OCV; <2% thought protection lasted 3–5 years, and 16% in Petite Anse and 10% in Cerca Carvajal thought protection lasted a lifetime.

### Adverse Events following Vaccination and Reasons for Nonvaccination

Among respondents who reported having received at least 1 dose of OCV, minor adverse events following the first dose were reported by 8% and following the second dose by almost 5%. The most commonly reported adverse events were nausea, vertigo, and abdominal pain (Table 3). No major adverse events were reported. The most common reason for not receiving the first or the second dose in both regions was absence during the campaign (Table 4).

**Table 3:** Adverse events reported within 14 days of receipt of oral cholera vaccine, by area, Haiti, 2013*

Adverse event	Petite Anse, no. (%)	Cerca Carvajal, no. (%)
First dose		
No. who received dose	768	691
Total events reported	68 (7.9; 95% CI 6.0–10.3)	56 (8.0; 95% CI 5.4–11.7)
Common events reported†		
Nausea	20 (2.6)	17 (2.5)
Vertigo	15 (2.0)	11 (1.6)
Abdominal pain	13 (1.7)	17 (2.5)
Weakness/fatigue	11 (1.4)	4 (0.6)
Diarrhea	9 (1.2)	9 (1.3)
Vomiting	5 (0.7)	5 (0.7)
Bloating	3 (0.4)	7 (1.0)
Fever	8 (1.0)	5 (0.7)
Headache	2 (0.3)	4 (0.6)
Rash	4 (0.5)	Not reported
Second dose		
No. who received dose	697	637
Total events reported	35 (4.7; 95% CI 3.0–7.3)	29 (4.1; 95% CI 2.4–6.8)
Common events reported‡		
Vertigo	6 (0.9)	7 (1.1)
Nausea	7 (1.0)	5 (0.8)
Abdominal pain	6 (0.9)	9 (1.4)
Vomiting	1 (0.1)	2 (0.3)
Diarrhea	2 (0.3)	4 (0.6)
Fever	4 (0.6)	3 (0.5)
Weakness/fatigue	4 (0.6)	1 (0.2)
Headache	3 (0.4)	3 (0.5)
Rash	6 (0.9)	Not reported
Bloating	2 (0.3)	Not reported

*The categories for adverse events are not mutually exclusive as participants had the option to report multiple adverse events.  †Denominator incudes persons who received the first dose. Percentages are unweighted for the purpose of description only. ‡Denominator includes persons who received the second dose. Percentages are unweighted for the purpose of description only.

**Table 4:** Principal reasons for not receiving oral cholera vaccine, by area, Haiti, 2013*

Reason	Petite Anse, no. ( %)	Cerca Carvajal, no. ( %)
First dose		
No. who did not receive dose	348	117
Absent during the campaign	141 (40.5)	63 (53.8)
Did not hear about the vaccination activities	41 (11.8)	15 (12.8)
Busy/no time	34 (9.8)	7 (6.0)
Sick during the campaign	14 (4.0)	4 (3.4)
Didn’t think vaccination was important/necessary	20 (5.7)	2 (1.7)
Don’t think vaccines are safe/vaccines can harm	10 (2.9)	2 (1.7)
Vaccines not available	5 (1.4)	4 (3.4)
Didn’t know when or where to go	6 (1.7)	2 (1.7)
Clinic closed/vaccinator not there/vaccinator refused to vaccinate	6 (1.7)	2 (1.7)
Other	71 (20.4)	16 (13.7)
Second dose†		
No. who did not receive dose	73	54
Absent during second campaign	21 (28.8)	13 (24.1)
Clinic closed/vaccinator refused to vaccinate	5 (6.8)	1 (1.9)
Busy/no time	4 (5.5)	11 (20.4)
Bad experience/ adverse event after first dose	6 (8.2)	2 (3.7)
Did not know needed a second dose	3 (4.1)	1 (1.9)
Sick during the campaign	2 (2.7)	Not reported
Didn’t know when or where to go	Not reported	2 (3.7)
Forgot to go	2 (2.7)	1 (1.9)
Vaccines not available	1 (1.4)	7 (13.0)
Other	29 (39.7)	9 (16.7)

*Categories are mutually exclusive because respondents were allowed to give only 1 primary reason. Percentages are unweighted for the purpose of description only. †Of those who received the first dose.

## Discussion

We report OCV coverage, barriers to vaccination, and adverse events after the first government-implemented OCV campaign in Haiti. The overall rates of 2-dose OCV coverage in rural Cerca Carvajal (77%) and urban Petite Anse (63%) were lower than the reported administrative coverage. Potential explanations could be the inaccurate population denominators used to calculate administrative coverage because the most recent census data were for 2003. In addition, several persons came from other areas to receive the vaccine, especially in Petite Anse, a crowded urban area, leading to overestimation of administrative coverage. Furthermore, the splitting and delaying of the second round of vaccination in Petite Anse created some confusion among the population regarding the vaccination dates and could have contributed to the high dropout rates. Nevertheless, the 2-dose OCV coverage achieved in Haiti is considered acceptable because herd immunity after 2-dose coverage with Shanchol as low as 28% has been reported (*25*), and mathematical models have shown that cholera might be controlled in disease-endemic settings starting with 2-dose OCV coverage of 50% (*26*).

Our results are comparable to those reported after a pilot OCV vaccination campaign conducted in 2012 by 2 NGOs in Haiti, for which coverage was 77% in rural Bocozel (*12*) and 69% in urban slums in Port-au-Prince (*13*). Two-dose OCV coverage rates in rural Haiti are similar to those reported in Bangladesh (72%) during the cholera off season (*11*) and rural Guinea (76%) during a cholera outbreak (*18*). Furthermore, OCV coverage rates in Haiti are among the highest observed thus far, compared with those reported after NGO-implemented campaigns in South Sudan, India, Mozambique, and Zanzibar (*9*,*10*,*14*,*15*). This campaign is one of the few OCV campaigns implemented by a government in a cholera-endemic setting; when the governments of Vietnam and Micronesia conducted OCV vaccinations in disease-endemic or outbreak settings, coverage rates were <80% and 50%, respectively (*16*,*17*).

OCV coverage was much lower among persons >15 years of age in both regions and lower among male than female respondents in Cerca Carvajal. Similar findings have been reported for Mozambique, Bangladesh, India, South Sudan, Guinea, and Vietnam (*9*,*11*,*14*,*15*,*18*,*27*). However, as in other countries (*8*,*9*,*12*,*18*,*28*), awareness and acceptance of OCV was relatively high. The major reason for not receiving the vaccine was absence during the campaign. Unlike previous vaccination campaigns in Haiti, which primarily targeted children or women of reproductive age, OCV campaigns targeted all nonpregnant persons >1 year of age. Vaccination campaigns focused on adults need to include vaccination sessions either very early in the morning or in the evenings, when working men and women are more likely to be at home. More than two thirds of persons in Petite Anse were vaccinated at home. Vaccinators visited homes during the day, when several respondents might have been at work or at the market. In addition, adults in general and men in particular may believe that vaccines are intended for children and might not seek vaccination. Hence, additional efforts are needed to explain the need for adults to receive OCV. If vaccines are available, additional activities to reach those who were not vaccinated because of absence during the campaign might help increase coverage.

Most respondents did not know the duration of protection provided by the vaccine, and almost one third thought that vaccine alone would be enough to protect them from cholera. This poor knowledge about the vaccine may result from limited messaging about the vaccine during the campaign; most of the information was spread through pamphlets, which probably were of limited usefulness because of low literacy rates in the target communities. These findings are concerning because persons who believe they are completely protected from cholera after vaccination might abandon protective behavior such as treating drinking water and practicing good hygiene. OCV campaigns should offer an opportunity to promote hygiene and safe water and food practices; an OCV campaign conducted in 2012 by an NGO in rural Haiti included a strong cholera and WASH education component and was associated with improved cholera knowledge and hygiene practices (*29*). Future campaigns in Haiti should focus on word-of-mouth messaging to spread cholera prevention educational information. Health care workers and trained social mobilizers with megaphones could transmit these messages before, during, and after the vaccination campaign.

Although the 200,000 OCV doses for the 2013 campaign were purchased directly from the manufacturer by the United Nations Children’s Fund, future OCV for use in cholera-endemic and -epidemic settings will be mainly obtained through the OCV stockpile managed by the International Coordinating Group and the Global Task Force on Cholera Control (*30*–*32*). Given the limited amount of vaccine available, epidemiologic, technical, and operational evidence, as well as local capacity to conduct OCV campaigns, will be assessed for optimal stockpile vaccine use. Moreover, the International Coordinating Group highlights the need to integrate OCV use with early case detection, appropriate case management, provision of adequate WASH infrastructure, and raising of awareness in the affected communities. Therefore, the cornerstones for cholera prevention and control remain safe water, improved sanitation, and adequate hygiene; WHO recommends that OCV use should complement traditional cholera control measures, including WASH interventions (7).

As has been noted for other Shanchol campaigns (*12*,*13*,*15*,*18*,*33*), no major adverse events were reported. Although the rates of minor adverse events were not higher than those reported by the manufacturer and in Bangladesh (*33*), they were higher than adverse events reported within 48 hours of vaccination during 2 pilot NGO-run OCV campaigns in Haiti (0.5%–1.3%) (*12*,*13*) and a campaign in Guinea (1%) (*18*).

This survey has 2 main limitations. Only half of respondents who received both OCV doses in Petite Anse and 70% of those in Cerca Carvajal could document OCV vaccination by card. Therefore, in some instances we based vaccine status on a patient’s verbal report, which could have led to overestimation of vaccine coverage. However, the extent of this bias was probably limited because the survey was conducted shortly after the campaign, and respondents correctly identified campaign dates and route of OCV administration. Second, overestimating coverage in the sample size calculation and underestimating the design effect in Cerca Carvajal contributed to the wide confidence intervals. However, we have reported the estimated design effect caused by clustering and the estimated intraclass correlation (Table 2, footnote). Future OCV surveys in Haiti can use these intraclass correlations, along with the expected number of responses in each cluster, to estimate the design effect caused by clustering.

In conclusion, coverage rates after the first government-implemented OCV campaign in Haiti were acceptable. As part of the national plan for the elimination of cholera, results from this survey would be essential for planning future OCV campaigns in Haiti to reach those who remain nonvaccinated. Given the lack of accurate data about target population estimates and high vaccine demand from nearby areas, it may be useful to overestimate required vaccine doses to improve vaccination coverage and avoid running out of vaccine during future campaigns. Printing enough vaccination cards and emphasizing the value of keeping the card and bringing it back when receiving the second vaccine dose are needed. Vaccination sessions should be tailored to reach persons who work during the day and men in general. Furthermore, OCV campaigns should be coordinated with WASH activities to ensure a comprehensive approach to cholera control and prevention and to promote the elimination of cholera from Haiti.
